# Supplementary figures and images for: Switching of Receptor Binding Poses between Closely Related Enteroviruses
Source: Viruses. 2022 Nov 24;14(12):2625. doi: 10.3390/v14122625 (PMC9781616; doi:10.3390/v14122625)

a

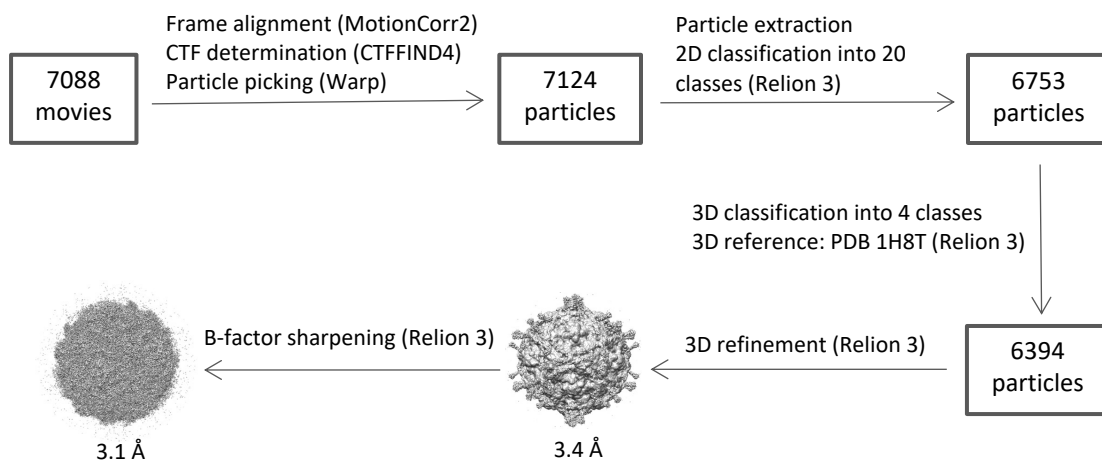

b

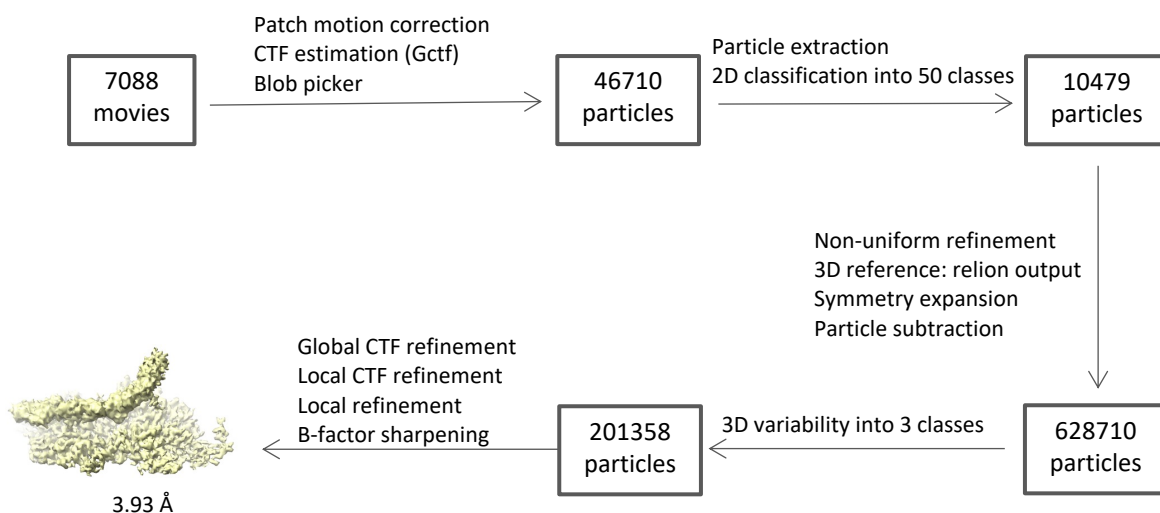

**Figure S1.** CryoEM processing workflows. (a) Relion, (b) Cryosparc.

Supplement: Supplementary file 1 [file viruses-14-02625-s001.zip › viruses-1981378-supplementary.pdf]
